# MEF2A Regulates the MEG3-DIO3 miRNA Mega Cluster-Targeted PP2A Signaling in Bovine Skeletal Myoblast Differentiation

**DOI:** 10.3390/ijms20112748

**Published:** 2019-06-04

**Authors:** Yaning Wang, Chugang Mei, Xiaotong Su, Hongbao Wang, Wucai Yang, Linsen Zan

**Affiliations:** 1 College of Animal Science and Technology, Northwest A&F University, Yangling 712100, Shaanxi, China; wangyn1992@outlook.com (Y.W.); meichugang@163.com (C.M.); xiaotongsu86@gmail.com (X.S.); wanghongbao@nwsuaf.edu.cn (H.W.); yangwucai111@163.com (W.Y.); 2 National Beef Cattle Improvement Center in China, Yangling 712100, Shaanxi, China

**Keywords:** MEF2A, MEG3-DIO3 miRNA cluster, PP2A signaling, bovine, myoblast differentiation

## Abstract

Understanding the molecular mechanisms of skeletal myoblast differentiation is essential for studying muscle developmental biology. In our previous study, we reported that knockdown of myocyte enhancer factor 2A (MEF2A) inhibited myoblast differentiation. Here in this study, we further identified that MEF2A controlled this process through regulating the maternally expressed 3 (MEG3)—iodothyronine deiodinase 3 (DIO3) miRNA mega cluster and protein phosphatase 2A (PP2A) signaling. MEF2A was sufficient to induce *MEG3* expression in bovine skeletal myoblasts. A subset of miRNAs in the MEG3-DIO3 miRNA cluster was predicted to target PP2A subunit genes. Consistent with these observations, MEF2A regulated PP2A signaling through its subunit gene protein phosphatase 2 regulatory subunit B, gamma (*PPP2R2C*) during bovine myoblast differentiation. MiR-758 and miR-543 in the MEG3-DIO3 miRNA cluster were down-regulated in MEF2A-depleted myocytes. Expression of miR-758 and miR-543 promoted myoblast differentiation and repressed *PPP2R2C* expression. Luciferase activity assay showed that *PPP2R2C* was post-transcriptionally targeted by miR-758 and miR-543. Taken together, these results reveal that the MEG3-DIO3 miRNAs function at downstream of MEF2A to modulate PP2A signaling in bovine myoblast differentiation.

## 1. Introduction

Skeletal muscle is an important constituent in maintaining metabolic homeostasis [1]. Vertebrate skeletal muscle development is a complex process that is controlled sequentially by somite commitment into progenitors, myoblasts proliferation, migration, fusion, and final adaptation into fast-twitch and slow-twitch muscle fibers [2]. During myogenesis, the cascade control of multiple transcription factors provides considerable effects [3,4].

Myocyte enhancer factor 2A (MEF2A), a basic helix-loop-helix (bHLH) transcription factor, which is highly expressed in the brain, heart, and skeletal muscle, is required for embryonic development, cardiomyocyte proliferation, skeletal muscle differentiation, and regeneration [5,6,7]. In mice, MEF2A-knockedout myoblasts are unable to differentiate and cause severe disruption of skeletal muscle regeneration, suggesting the key roles of MEF2A in skeletal muscle [8]. Similarly, our previous study shows that MEF2A is indispensable for bovine skeletal myoblast differentiation [9]. However, despite the findings that MEF2A participates in calcineurin, mitogen-activated protein kinase (MAPK), and Wnt signaling pathways in cell division and differentiation [8,10,11], a detailed understanding of MEF2A function in skeletal muscle is far from complete.

MicroRNAs (miRNAs) are small, non-coding RNAs that are widely distributed in various tissues and potentially participate in the regulation of almost all cellular processes investigated so far [12]. In skeletal muscle myogenesis, a limited number of miRNAs have been identified, including the muscle-specific miR-1 and miR-133a [13,14,15]. Interestingly, some recent studies have found that miRNAs in mouse Gtl2-Dio3 locus (corresponding to the bovine MEG3-DIO3 locus) appear to play important roles in regulating skeletal muscle regeneration [8,16]. The Gtl2-Dio3 locus is genomic imprinted and expresses the largest miRNA cluster in mammals [17]. These miRNAs are solely expressed from the maternally inherited allele [17]. It is reported that expression of these miRNAs is coordinately controlled by the adjacent *Gtl2* promoter [8,18,19]. Given the complexity of the Gtl2-Dio3 locus, the regulatory functions exerted by this gene cluster in skeletal muscle is not understood completely.

Protein phosphatase 2A (PP2A) is one of the main serine/threonine phosphatases in mammalian cells and acts as a negative regulator for several signaling pathways which promote cell proliferation, survival, signal transduction, and tumor progression [20,21,22]. The PP2A core enzyme is composed of a scaffolding A subunit and a catalytic C subunit. To gain full activity toward specific substrates, the PP2A core enzyme interacts with a regulatory B subunit [23]. The regulatory B subunit is a key mediator in modulating PP2A activity by targeting a wide range of PP2A substrates [24]. In skeletal muscle myogenesis, proteins such as ubiquitin and phosphorylation undergo dramatic post-translational modifications [25]. Although PP2A plays crucial roles in multiple cellular processes, at present it remains completely unknown whether and how PP2A regulates myogenesis.

We previously reported that MEF2A was essential for skeletal myoblast differentiation. Inhibition of MEF2A resulted in severely disrupted myotube formation [9]. Here in this study, we further investigated the roles of MEF2A in regulating myogenesis. We found that MEF2A controlled bovine myoblast differentiation through maternally expressed 3 (MEG3) - iodothyronine deiodinase 3 (DIO3) miRNA cluster which further targeted the PP2A signaling pathway. These findings establish a novel molecular role for the MEF2A-MEG3/DIO3-PP2A axis in myogenesis and may shed new light on the developmental mechanisms of skeletal muscle.

## 2. Results

### 2.1. Myocyte Enhancer Factor 2A (MEF2A) Is Sufficient to Induce Maternally Expressed 3 (MEG3) Expression

**Figure 1:**
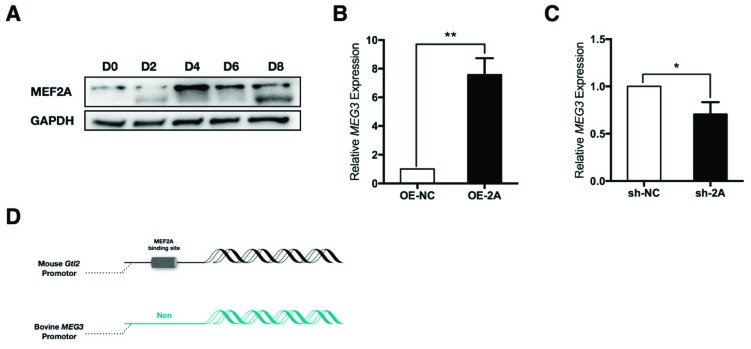
Myocyte enhancer factor 2A (MEF2A) is sufficient to induce maternally expressed 3 (*MEG3*) expression. (**A**) Time course of MEF2A protein expression from differentiation day 0 (D0) to differentiation day 0 (D8) of myoblasts; (**B**) Overexpression of MEF2A obviously induced *MEG3* expression in differentiated myocytes; (**C**) Inhibition of MEF2A repressed *MEG3* expression in differentiated myocytes; (**D**) Transcription factor binding motif prediction of the mouse *Gtl2* promotor and bovine *MEG3* promotor. Error bars represent standard error of the mean (SEM). “*” represents *p* < 0.05. “**” represents *p* < 0.01.

In our previous study, we reported that transcript expression of *MEF2A* was obviously induced during skeletal muscle development and myoblast differentiation [9]. Here in this study, we found that the total MEF2A protein expression level was also elevated during myoblast differentiation (Figure 1A). These results suggest that MEF2A is an essential inducer for myogenesis.

Previous studies reported that the mouse *Gtl2-Dio3* miRNA cluster had a close correlation with myoblast differentiation. MEF2A activated the transcription of these miRNAs through binding to the MEF2 motif on the *Gtl2* promoter [8]. To test this role in bovine skeletal myoblasts, we firstly detected the expression correlation between MEF2A and *MEG3*. We found that the expression of MEF2A obviously up-regulated *MEG3* expression, and inversely, the interference of MEF2A reduced *MEG3* expression (Figure 1B,C). To explore whether MEF2A regulated *MEG3* expression through directly activating the *MEG3* promoter, first of all, transcription factor binding motifs were analyzed by using online MatInspector software. Surprisingly, unlike the mouse *Gtl2* promoter, there were no potential MEF2A binding sites identified on the bovine *MEG3* promoter (Figure 1D). Given the complex regulation of MEF2A in skeletal muscle, it is supposed that MEF2A might regulate bovine *MEG3* expression through interacting with other proteins [26].

### 2.2. Identification of Predicted Targets of the Bovine MEG3 - Iodothyronine Deiodinase 3 (DIO3) miRNA Cluster

**Figure 2:**
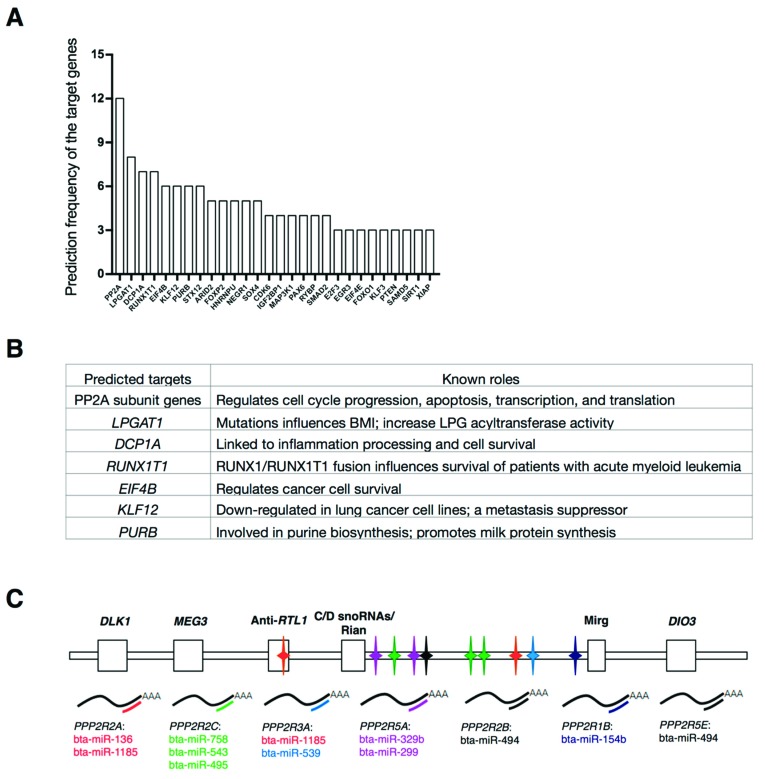
Identification of predicted target genes of the MEG3- iodothyronine deiodinase 3 (DIO3) miRNA cluster. (**A**) Prediction frequency of the genes potentially targeted by the miRNAs in the MEG3-DIO3 locus; (**B**) The top seven predicted targets of the MEG3-DIO3 miRNA cluster and their known roles in mammalian cells; (**C**) Target prediction diagrams of protein phosphatase 2A (PP2A) subunit genes by the MEG3-DIO3 miRNA cluster. A total of one scaffolding subunit gene and six regulatory subunit genes were predicted.

In bovine MEG3-DIO3 locus, there were two protein-cording genes, seven long-noncoding RNAs (lncRNAs) and fifty-six miRNAs in this locus (Table S1). To identify the potential regulatory networks targeted by the MEG3-DIO3 miRNA cluster, all of the miRNAs in this cluster were introduced to miRNA-mRNA prediction using TargetScan (Table S2). The results showed that, among all the potential targets, dozens of genes that played essential roles were frequently predicted (Figure 2A). These targets, including PP2A enzyme subunit genes, lysophosphatidylglycerol acyltransferase 1 (*LPGAT1*), decapping mRNA 1A (*DCP1A*), RUNX family transcription factor 1 translocation partner 1 (*RUNX1T1*), eukaryotic translation initiation factor 4B (*EIF4B*), kruppel like factor 12 (*KLF12*), and purine rich element binding protein B (*PURB*), mainly function in bioprocesses including cell growth, apoptosis, inflammation, lipid metabolism, and neuron biology (Figure 2B) [22,23,27,28,29,30,31,32,33,34]. Among these potential targets, the most frequently predicted genes were PP2A subunit genes which had been predicted over 12 times (Figure 2A). PP2A is an essential protein phosphatase in regulating cell cycle progression, apoptosis, transcription, and translation [22,23]. Consequently, we focused on PP2A to investigate the roles of the MEG3-DIO3 miRNA cluster in myogenesis. 

In this study, according to the miRNA-mRNA prediction, one gene of protein phosphatase 2 scaffold subunit A, beta (*PPP2R1B*) that encoded subunit A protein and six genes including protein phosphatase 2 regulatory subunit B, alpha/beta/gamma (*PPP2R2A*, *PPP2R2B*, *PPP2R2C)* protein phosphatase 2 regulatory subunit B′ alpha/epsilon (*PPP2R5A*, *PPP2R5E*), and protein phosphatase 2 regulatory subunit B″ alpha (*PPP2R3A*) that encoded subunit B proteins, were predicted by ten miRNAs in the MEG3-DIO3 miRNA cluster (Figure 2C). These results imply that MEF2A might regulate myoblast differentiation through modulating MEG3-DIO3 miRNAs-targeted PP2A signaling. To verify this hypothesis, a series of three experiments should be performed: (a) MEF2A surely regulates PP2A signaling in myoblast differentiation; (b) MEF2A modulates MEG3-DIO3 miRNAs in myoblast differentiation; (c) MEG3-DIO3 miRNAs target PP2A signaling.

### 2.3. MEF2A Regulates Protein Phosphatase 2A (PP2A) Signaling During Myoblast Differentiation

**Figure 3:**
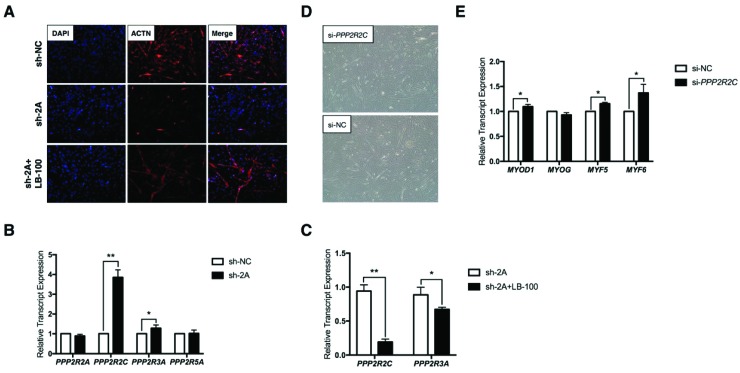
MEF2A regulates PP2A signaling during myoblast differentiation. (**A**) Inhibition of MEF2A repressed myoblast differentiation, while the addition of LB-100 rescued the disrupted myotube formation (magnification: 100×); (**B**) Real time PCR (RT-PCR) analysis of *PPP2R2A*, *PPP2R2C*, *PPP2R3A*, and *PPP2R5A* in MEF2A-inhibited myocytes; (**C**) RT-PCR analysis of *PPP2R2C* and *PPP2R3A* in the presence of LB-100 in MEF2A-silienced myocytes; (**D**) Effects of *PPP2R2C* silence on myoblast differentiation (magnification: 40×); (**E**) RT-PCR analysis of *MYOD1*, *MYOG*, *MYF5*, and *MRF6* in *PPP2R2C*-silenced myocytes. Error bars represent SEM. “*” represents *p* < 0.05. “**” represents *p* < 0.01.

To explore the roles of PP2A signaling in myoblast differentiation, PP2A inhibitor LB-100 rescue assay was performed. As shown in Figure 3A, the results revealed that PP2A signaling absolutely participated in MEF2A regulated myoblast differentiation. Interference of MEF2A severely inhibited myogenesis as observed by the immunostaining of actinin alpha (ACTN), the myogenic differentiation marker. Surprising, when LB-100 was added, the impaired myoblast differentiation was nearly completely rescued. Based on the prediction frequency (Figure 2C), *PPP2R2A*, *PPP2R2C*, *PPP2R3A*, and *PPP2R5A* were subjected to further analyses. Real time PCR (RT-PCR) analysis showed that the expression of *PPP2R2C* and *PPP2R3A*, except for *PPP2R2A* and *PPP2R5A*, were up-regulated in MEF2A-depleted myoblasts (Figure 3B). In particular, the mRNA expression of *PPP2R2C* was sharply elevated nearly four times as much as the control group (Figure 3B). Moreover, in this rescue assay, LB-100 efficiently inhibited expression of *PPP2R2C* and *PPP2R3A* (Figure 3C), indicating that *PPP2R2C* and *PPP2R3A* might have pivotal roles in the MEF2A-regulated PP2A signaling pathway during myoblast differentiation. Due to the strong effects of MEF2A and LB-100 on *PPP2R2C* expression, *PPP2R2C* was subjected to further analysis.

In skeletal muscle, four myogenic regulatory factors (MRFs) including myogenic differentiation 1 (MYOD1), myogenin (MYOG), myogenic factor 5 (MYF5), and myogenic factor 6 (MYF6) act together to modulate myoblast differentiation [2]. MYF5 induces the myogenic commitment of progenitor cells and highly expresses in the proliferating myoblasts. MYOD1 activates the myogenic determination and early differentiation. MYF6 and MYOG regulate the late differentiation of myogenesis [4]. To investigate the role of *PPP2R2C* in myoblast differentiation, *PPP2R2C* silencing assay was performed by using specific short interference RNA (siRNA). As shown in Figure 3D, inhibition of *PPP2R2C* promoted myotube formation to some extent. At the transcriptional level, *MYOD1*, *MYF5*, and *MYF6* except for *MYOG* were all up-regulated in *PPP2R2C*-silienced myocytes (Figure 3E). These results indicate that *PPP2R2C* is an inhibitor of myogenic differentiation and mainly regulates the myogenic commitment and early differentiation stage. 

### 2.4. MEF2A Regulates MEG3-DIO3 miRNAs During Myoblast Differentiation

**Figure 4:**
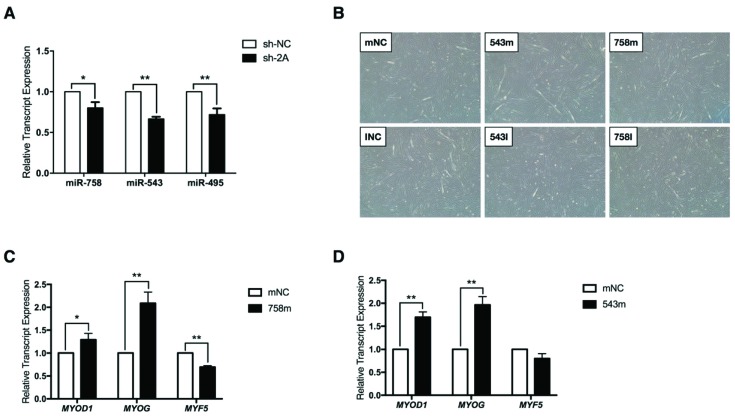
MEF2A regulates MEG3-DIO3 miRNAs during myoblast differentiation. (**A**) RT-PCR analysis of miR-758, miR-543, and miR-495 in MEF2A-depleted myocytes; (**B**) Overexpression and inhibition effects of miR-758 and miR-543 on myoblast differentiation (magnification: 40×); (**C**) RT-PCR analysis of *MYOD1*, *MYOG*, and *MYF5* upon miR-758 mimics transfection during myoblast differentiation; (**D**) RT-PCR analysis of *MYOD1*, *MYOG*, and *MYF5* upon miR-543 mimics transfection during myoblast differentiation. Error bars represent SEM. “*” represents *p* < 0.05. “**” represents *p* < 0.01.

Because miR-758, miR-543, and miR-495 were the miRNAs that might target *PPP2R2C* (Figure 2C), in this study we investigated whether and how these three miRNAs affected myoblast differentiation. RT-PCR analysis showed that the expression of miR-758, miR-543, and miR-495 were down-regulated in MEF2A-depleted myoblasts (Figure 4A). The transfection of miR-758 mimics (758 m) and miR-543 mimics (543 m) obviously promoted myoblast differentiation compared with the mimic negative control (mNC) group (Figure 4B). Inversely, in comparison with the inhibitor negative control (INC) group, miR-758 inhibitor (758I) and miR-543 inhibitor (543I) repressed myoblast differentiation (Figure 4B). However, miR-495 had no obvious effects on myoblast differentiation. Consistent with these observations, RT-PCR analysis showed that transfection of miR-758 mimics and miR-543 mimics significantly up-regulated *MYOD1* and *MYOG* expression (Figure 4C,D). miR-758 mimics also inhibited expression of *MYF5* which is usually highly expressed in undifferentiated myoblasts (Figure 4C). Taken together, these results suggest that MEF2A surely regulates MEG3-DIO3 miRNAs during myoblast differentiation. Given that the sharp elevation of *PPP2R2C* expression induced by MEF2A was predicted by miR-758, miR-543, and miR-495 (Figure 2C and Figure 3B), we questioned whether *PPP2R2C* was regulated by the MEG3-DIO3 miRNA cluster.

### 2.5. Protein Phosphatase 2 Regulatory Subunit B, Gamma (PPP2R2C) Expression Is Inhibited by miR-758 and miR-543

**Figure 5:**
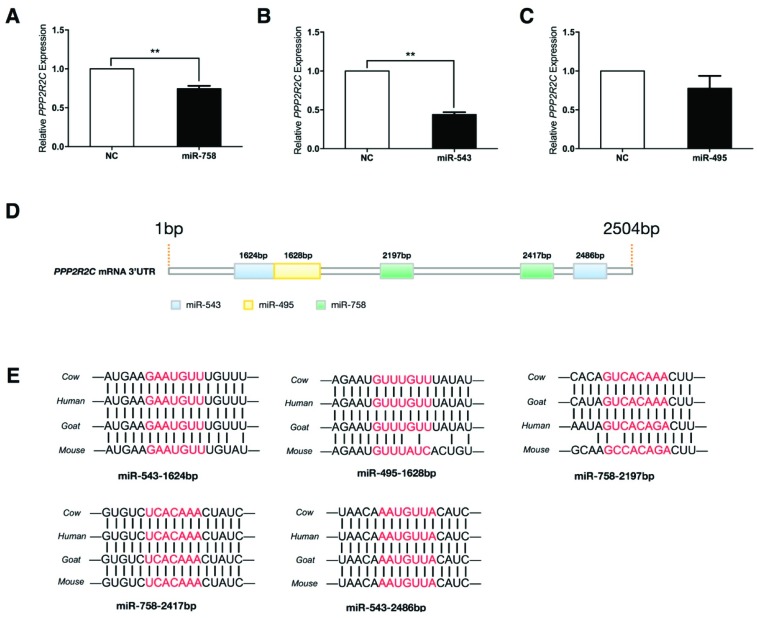
Protein phosphatase 2 regulatory subunit B, gamma (*PPP2R2C*) expression is inhibited by miR-758 and miR-543. (**A**–**C**) RT-PCR analysis of *PPP2R2C* in the presence of miR-758 mimics, miR-543 mimics, and miR-495 mimics in differentiated myocytes; (**D**) Diagrams of the potential miR-758, miR-543, and miR-495 binding sites in the 3′ untranslated region (3′UTR) of *PPP2R2C* mRNA; (**E**) Sequence alignments of the five miRNA binding sites among mammals. Error bars represent SEM. “**” represents *p* < 0.01.

On the basis of the results obtained in this study, next the expression correlations between *PPP2R2C* and the three miRNAs were investigated. RT-PCR analysis showed that *PPP2R2C* expression was down-regulated in the presence of miR-758 mimics and miR-543 mimics (Figure 5A,B), while miR-495 had no effects on *PPP2R2C* expression (Figure 5C). To explore whether these miRNAs modulated *PPP2R2C* expression through post-transcriptional regulation, the 3′ untranslated region (3′UTR) of *PPP2R2C* mRNA was subjected to detailed analysis. As shown in Figure 5D, two miR-543 binding sites, two miR-758 binding sites, and one miR-495 binding site were predicted in the *PPP2R2C* 3′UTR. Subsequently, sequence alignments of the five miRNA binding sites were analyzed to determine the sequence conservation among mammals. The results showed that the miRNA binding sites of miR-543-1624bp, miR-758-2417bp, and miR-543-2486bp were completely conserved among cows, humans, goats, and mice (Figure 5E). The other two miRNA binding sites including miR-495-1628bp and miR-758-2197bp were less conserved when compared the transcriptome database between cows and mice (Figure 5E). Notably, among the five miRNA binding sites, miR-543-1624bp and miR-495-1628bp had an overlap of three nucleotides (GUU, red letters in Figure 5E), suggesting that in this region, there might be only one miRNA that could sufficiently bind to *PPP2R2C* mRNA. Taken together, these findings indicate that *PPP2R2C* might be post-transcriptionally regulated by miR-758, miR-543, and miR-495.

### 2.6. PPP2R2C Is a Direct Target of miR-758 and miR-543

**Figure 6:**
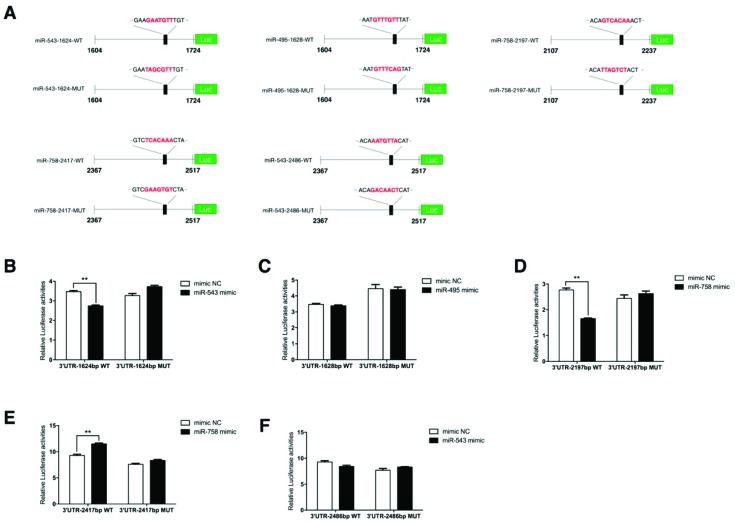
*PPP2R2C* is a direct target of miR-758 and miR-543. (**A**) Construction of the luciferase reporter vectors for 3′UTR of *PPP2R2C* mRNA containing wild type (WT) or mutant (MUT) miRNA binding sites. The seed sequences of the WT and MUT miRNA binding sites are marked in red; (**B**–**F**) Luciferase reporter activity assay of *PPP2R2C* mRNA 3′UTR containing the five potential miRNA binding sites. Error bars represent SEM. “**” represents *p* < 0.01.

To investigate whether *PPP2R2C* was a downstream target of miR-758, miR-543, and miR-495, luciferase activity assays were introduced by using the psiCHECK-2 reporter vector. Ten reporter vectors containing the wide type (WT) or mutant (MUT) miRNA binding sites were generated and transfected together with the corresponding miRNA mimics in 293T cells (Figure 6A). The mutant miR-543-1624 and miR-495-1628 reporters were generated by mutating GAAT to TAGC and TGTT to TCAG, respectively, without altering the overlapping nucleotides GTT. Luciferase activity assays showed that three out of the five potential miRNA binding sites interacted with the miRNAs (Figure 6B–F). Activities of the WT 3′UTR-1624bp vector and the WT 3′UTR-2197bp vector were down-regulated in the presence of miR-543 mimics (Figure 6B) and miR-758 mimics (Figure 6D), respectively, while activity of the WT 3′UTR-2417bp vector was elevated in the presence of miR-758 mimics (Figure 6E). It is possible that this up-regulating effect might be achieved by unconventional miRNA functions [35]. For the other two remaining vectors containing the predicted miRNA binding sites 3′UTR-1628bp and 3′UTR-2486bp, miR-495 and miR-543 were not capable of regulating their activities (Figure 6C,F). These results suggest that *PPP2R2C* is a direct target of the MEG3-DIO3 miRNA cluster that can be regulated by miR-758 and miR-543.

**Figure 7:**
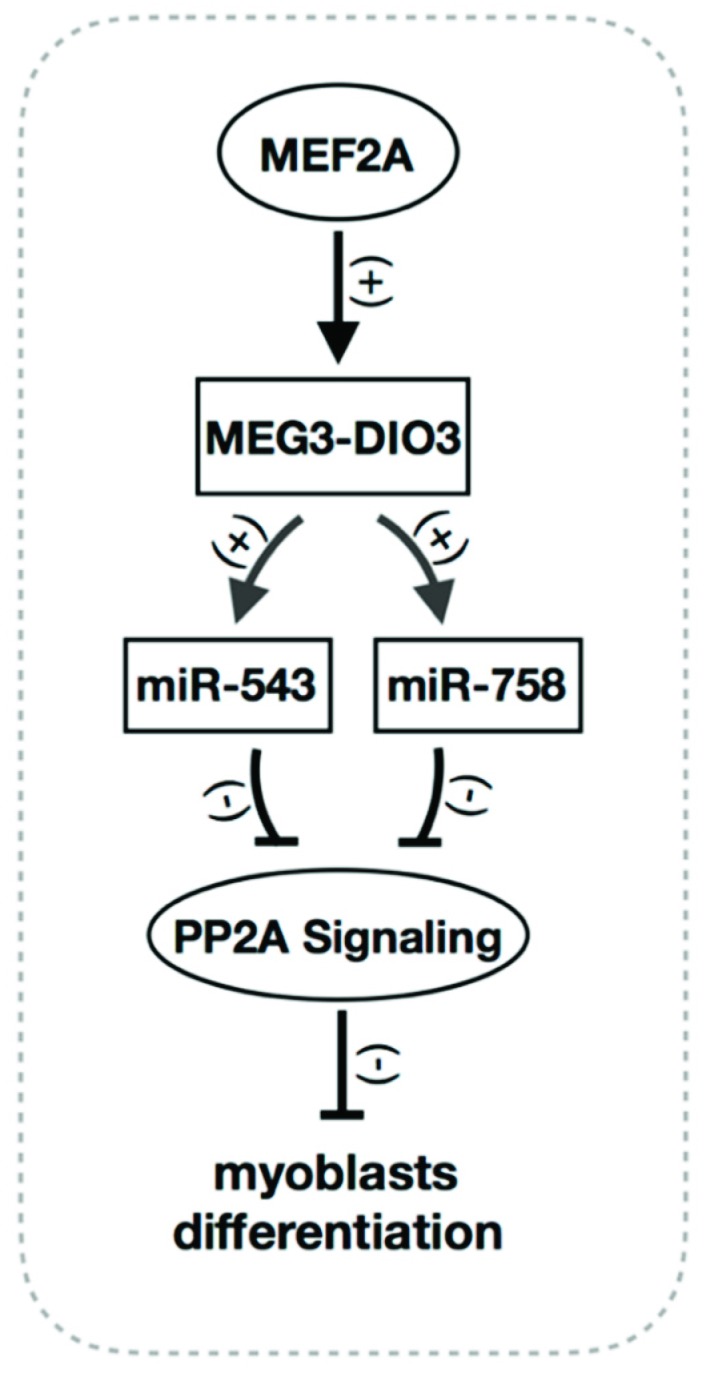
The potential roles of MEF2A-MEG3/DIO3-PP2A signaling regulatory axis in myoblast differentiation. That is, the MEG3-DIO3 miRNA cluster functions downstream of MEF2A to modulate PP2A signaling in myoblast differentiation. Black arrows and “(+)” represent positive regulation. T-bars and “(−)” represents negative regulation.

Taken together, our study firstly demonstrates a connection between MEF2A and PP2A signaling in myoblast differentiation. This study reveals that the MEG3-DIO3 miRNA cluster functions downstream of MEF2A to modulate PP2A signaling in myoblast differentiation (Figure 7).

## 3. Discussion

In our previous study, we reported that MEF2A was required for skeletal myoblast differentiation. MEF2A depletion in differentiating myocytes severely inhibited myotube formation [9]. Here in this study, our results established a novel molecular role for MEF2A to regulate the MEG3-DIO3 miRNA cluster and PP2A signaling in skeletal myoblast differentiation. To our knowledge, this is the first evidence that PP2A signaling is involved in MEF2A-regulated skeletal muscle development.

Skeletal muscle development is a complex process which is coordinated with dramatic alteration of protein phosphorylation [25]. During myogenesis, a sustained elevation of intracellular free calcium activates calcineurin, a calcium/calmodulin-dependent serine/threonine phosphatase [36]. As a transcription activator in myocytes, MEF2A activity is stimulated by the dephosphorylation of the calcium-activated, calcineurin-dependent mechanism [36]. In this study, we found that total MEF2A protein was elevated during myoblast differentiation. Meanwhile, two protein bands with different molecular weights of MEF2A were detected on the sodium dodecyl sulfate-polyacrylamide gel electrophoresis (SDS-PAGE) gel. According to what Wu et al. reported, the upper band was the phosphorylated form and the lower band was the dephosphorylated form of MEF2A [36]. During myogenesis, the phosphorylation of MEF2A was attenuated and the dephosphorylation of MEF2A was enhanced, indicating that MEF2A activity was elevated. It is postulated that the trans-activation of MEF2A protein by dephosphorylation is the key in regulating myoblast differentiation. This is also approved by our previous study reporting that the overexpression of MEF2A results in the enrichment of numerous dephosphorylated proteins [9].

The MEF2A protein contains a highly conserved MCM1, Agamous, Deficiens, Serum Response factor (MADS) domain at its N-termini that mediates dimerization and binding to the DNA sequences. Adjacent to the MADS domain is a MEF2 domain that influences DNA-binding affinity and cofactor interactions [10,37]. The mouse *Gtl2-Dio3* miRNA cluster was firstly reported to be regulated by MEF2A in muscle regeneration by Snyder et al. [8]. They demonstrated that MEF2A directly regulated the mouse *Gtl2* promoter upstream of all miRNAs in the cluster by binding to the −39 bp MEF2 binding site. However, this hypothesis was challenged by another study suggesting that MEF2A did not depend on an intact DNA binding domain to stimulate expression of the *Gtl2-Dio3* miRNA cluster, because a MADS domain-truncated MEF2A isoform still efficiently induces expression of the *Gtl2-Dio3* miRNA cluster [16]. In our study, we found a positive regulation of MEF2A on the expression of *MEG3* and miRNAs within the MEG3-DIO3 gene cluster. However, through transcription factor binding motif prediction by Genomatix software, no potential MEF2A binding sites were identified on the bovine *MEG3* promoter. In such a situation, it is hard to verify the interactions between MEF2A and MEG3 through electrophoretic mobility shift assays or chromatin immunoprecipitation-PCR. Since MEF2A activates its target genes relying on the recruitment of, and cooperation with, other transcription factors [37,38], it is possible that the regulation of MEF2A on *MEG3* expression is likely due to interacting with other transcription factors.

In this study, TargetScan release 6.2 (2012 to 2015) and 7.2 (2016 to 2018) were used to predict the target genes of the bovine MEG3-DIO3 miRNA cluster. There were two reasons why we selected such approaches: (1) Among the frequently-used prediction algorithms, including miRanda, TargetScan, and miRDB, only TargetScan contains a database for cows; (2) in this study, the prediction analysis was initially performed in 2015 by using release 6.2. When the latest version was updated in 2018, the MEG3-DIO3 miRNA cluster was subjected to prediction again by using release 7.2. The release 7.2 developed an improved quantitative model of canonical targeting to minimize confounding biases and predict the most effectively targeted mRNAs [39]. In the present study, each miRNA was predicted twice by using the two databases and the commonly predicted genes were regarded as potential targets. What should be noted is that, for some miRNAs, only few mRNAs are commonly predicted by the two databases (Table S3). Thus, it is necessary to combine different prediction algorithms when performing miRNA—mRNA prediction analysis.

It is intriguing that PP2A subunit genes emerge as the top predicted targets by the MEG3-DIO3 miRNA cluster and *PPP2R2C* is directly targeted by miR-758 and miR-543. This is the first evidence that PP2A participates in MEF2A-regulated myoblast differentiation. PP2A holoenzyme contains three subunits (A, B, and C), and each subunit is encoded by various genes [22]. The diversity of PP2A derives from the fact that over 200 PP2A complexes can be assembled due to different interactions and combinations of the three subunits with other proteins [40]. The regulatory subunit is the key to mediate PP2A activity. In different mammalian tissues, cells express different regulatory subunit proteins at different developmental stages [41]. However, it is unknown how PP2A activities occur in skeletal muscle and whether there are specific subunit proteins regulating skeletal muscle development. In our present study, we find that PP2A signaling is under the control of MEF2A, and absolutely participates in myoblast differentiation. Meanwhile, *PPP2R2C* plays essential roles in MEF2A-regulated myoblast differentiation. It is confirmed that PP2A signaling is a negative regulator in modulating myogenesis, which is consistent with its dephosphorylation function to its substrates.

Given the identification of the MEF2A-MEG3/DIO3-PP2A signaling cascade regulation in myoblast differentiation, it is interesting to speculate that this regulatory pathway also plays an important role in skeletal muscle regeneration. MEF2A is an important activator in muscle regeneration [5,8]. Previous researches have illustrated the role of the MEF2A-*Gtl2/Dio3*-WNT pathway in regulating mouse muscle regeneration [8]. In this study, we introduced the PP2A signaling to MEF2A-MEG3/DIO3 axis-regulated myoblast differentiation, which will provide new insights into the molecular mechanisms of skeletal muscle myogenesis.

## 4. Materials and Methods

### 4.1. Cell Culture

Bovine primary skeletal myoblasts were isolated as described previously [9]. The experiments were performed in accordance with the guiding principles for the experimental animals adopted by the Ministry of Science and Technology. Animal care was approved by the Institutional Animal Care and Use Committee of Northwest A&F University (approved code: No.142, 13 June, 2014). Myoblasts were cultured in DMEM/F-12 (Gibco, Shanghai, China) supplemented with 20% fetal bovine serum (FBS, Gibco) and 1% penicillin/streptomycin (Hyclone, ThermoFisher Scientific, Shanghai, China). For myogenic differentiation, myoblasts were cultured in DMEM/F-12 supplemented with 2% horse serum (Gibco) and 1% penicillin/streptomycin. 293T cells were cultured in DMEM/High Glucose (Gibco) supplemented with 10% FBS and 1% penicillin/streptomycin. The cell culture medium was changed every two days.

### 4.2. Adenovirus, Vectors, and RNA Oligonucleotides

Adenoviruses carrying the full-length bovine *MEF2A* gene coding sequence (termed as OE-2A) and specific short hairpin RNA (shRNA) targeting *MEF2A* mRNA (termed as sh-2A) as well as the negative control adenoviruses (termed as OE-NC and sh-NC) were generated as described previously [9]. The ten reporter vectors were generated by cloning the 3′UTR containing the WT or MUT miRNA binding sites into the psiCHECK-2 vector (Promega, Beijing, China). miRNA mimics were used to increase the expression of miR-758, miR-543, and miR-495. miRNA inhibitors were used to reduce the expression of miR-758, miR-543, and miR-495. The RNA oligonucleotides of the three miRNAs and NC were chemically synthesized by Guangzhou RiboBio Co., Ltd. (Guangzhou, China).

### 4.3. Transfection and Luciferase Activity Assay

For the transfection of miRNA mimics in myoblasts, cells at 70% confluence were transfected with the miRNA oligonucleotides by using lipofectamine™ 3000 transfection reagent (Invitrogen, Shanghai, China) at a final concentration of 30 nM. For co-transfection of miRNA oligonucleotides and psiCHECK-2 reporter vectors, 293T cells were seeded in 24-well cell culture plates (Corning, Shanghai, China) in triplicate wells. For each treatment, 100 μg plasmids and 5 µL of 20 µM miRNA oligonucleotides were mixed and transfected into cells. Transfection was performed according to the manufacturer’s instructions.

The luciferase activity was measured 40 h after transfection by using the Dual-Luciferase Reporter Assay System (Promega) on Infinite1 200 PRO NanoQuant spectrophotometer (TECAN, Shanghai, China). The firefly luciferase activity was measured at 560 nm and the Renilla luciferase activity was measured at 480 nm. The relative activity of the reporter vector was determined as the ratio of renilla luciferase activity to firefly luciferase activity.

### 4.4. Western Blot Analysis

The protein samples were obtained from the differentiating myoblasts at differentiation day 0 (D0), differentiation day 2 (D2), differentiation day 4 (D4), differentiation day 6 (D6), and differentiation day 8 (D8). Protein concentration was measured using BCA method (TaKaRa, Beijing, China). For each sample, 20 μg denatured proteins were subjected to 12% SDS-PAGE gel and transferred to polyvinylidene fluoride (PVDF) membrane. The immune blot assay was performed as previously described [9]. The antibodies were used as follows: anti-MEF2A antibody [EP1706Y] (rabbit monoclonal primary antibody, 1:1000, abcam, Shanghai, China) and anti-GAPDH antibody [EPR16884] (rabbit monoclonal primary antibody, 1:10,000, abcam).

### 4.5. Quantitative Real Time-PCR

Total RNAs from the myocytes (*n* = 3) were isolated by using the RNAiso Plus kit (TaKaRa). RNA quantification was detected on Infinite1 200 PRO NanoQuant spectrophotometer. The cDNAs for mRNAs were reversely transcribed by using the Prime-Script^TM^ RT reagent Kit with gDNA Eraser (TaKaRa) and for miRNAs were synthesized by using the miRcute Plus miRNA First-Strand cDNA Synthesis Kit (TIANGEN, Beijing, China). Quantitative RT-PCR was performed in triplicate wells with the 7500 Real Time PCR System (Applied Biosystems, Shanghai, China). TB Green^TM^ Premix Ex Taq^TM^ II (Tli RNaseH Plus, TaKaRa) was used for protein-coding gene analysis and the miRcute Plus miRNA qPCR Detection Kit (SYBR Green, TIANGEN) was used for miRNA analysis. Glyceraldehyde-3-phosphate dehydrogenase (*GAPDH*) and U6 served as internal control for protein-cording genes and miRNAs respectively. Sequence information for the primers used in this study is provided in Table S3.

### 4.6. Cell Culture Immunofluorescence

Cells grown in 6-well cell culture plates were fixed with 4% paraformaldehyde for 15 min, rinsed with phosphate buffer solution (PBS) three times, permeabilized with 0.2% Triton X-100 (Sigma-Aldrich, Shanghai, China) for 15 min and then incubated in 10% normal donkey serum (Solarbio, Beijing, China)/1% BSA (Sigma-Aldrich)/0.3 M glycine (Sigma-Aldrich) for 1 h to block non-specific protein—protein interactions. The primary antibody was diluted in the blocking buffer to incubate cells overnight at 4 °C. After being washed with PBS three times, cells were incubated with secondary antibody protected from light at 37 °C for 1 h. The nuclei were stained with 4’,6-diamidino-2-phenylindole (DAPI) (Sigma-Aldrich) in the dark at room temperature for 10 min. The antibodies were used as follows: anti α-actinin (H-300) (1:200, Santa Cruz Biotechnology, Shanghai, China), and donkey anti-rabbit IgG H&L (Alexa Fluor^®^ 555, 1:1000, abcam). DAPI was used at the final concentration of 1 μg/mL. Phase-contrast and fluorescent microscopy were performed by using an inverted fluorescence microscope (Olympus IX71, Olympus Corporation, Beijing, China).

### 4.7. Computational Analysis

According to the National Center for Biotechnology Information database, bovine MEG3-DIO3 locus is located on chromosome 21 and contains 56 miRNAs. The potential target mRNAs of all the 56 miRNAs were predicted twice by using the TargetScan cow database release 6.2 and release 7.2. For each miRNA, the two subsets of the predicted mRNAs generated from the two databases were subjected to overlap diagram analysis. The commonly predicted mRNAs were regarded as potential targets of the corresponding miRNA. For each target gene, the prediction frequency was also analyzed. Transcription factor binding motif enrichment analysis for the mouse and bovine *MEG3* promoter was performed with MatInspector from the Genomatix software [7].

### 4.8. PP2A Inhibitor LB-100 Rescue Assay

Myoblasts were cultured in 6-well cell culture plates and infected with sh-2A and sh-NC at multiplicity of infection (MOI) 50. The PP2A inhibitor LB-100 (Selleck, Shanghai, China) was added 24 h post infection of adenoviruses at a final concentration of 1 µM. Myoblasts were induced to myogenic differentiation for 6 days before microscopy and RT-PCR analysis.

### 4.9. Statistical Analysis

All data were presented as mean ± SEM. Statistically significant differences between two groups were analyzed using independent-samples *t*-test (IBM SPSS Statistics version 18.0, IBM, Xi’an, China). *p* values of ≤ 0.05 were considered to be statistically significant.

## Supplementary Materials

Supplementary materials can be found at https://www.mdpi.com/1422-0067/20/11/2748/s1.

## Abbreviations

MEF2A	myocyte enhancer factor 2A
bHLH	basic helix-loop-helix
MAPK	mitogen-activated protein kinase
PP2A	protein phosphatase 2A
*PPP2R2C*	protein phosphatase 2 regulatory subunit B, gamma
MEG3	maternally expressed 3
DIO3	iodothyronine deiodinase 3
ACTN	actinin alpha
FBS	fetal bovine serum
OE-2A	adenovirus to overexpress MEF2A
OE-NC	negative control for OE-2A
sh-2A	adenovirus to expression MEF2A shRNA
sh-NC	negative control for sh-2A
D0	differentiation day 0
D2	differentiation day 2
D4	differentiation day 4
D6	differentiation day 6
D8	differentiation day 8
lncRNA	long-noncoding RNA
miRNA	microRNA
shRNA	short hairpin RNA
siRNA	short interference RNA
MADS	MCM1, Agamous, Deficiens, Serum Response factor
758m	miR-758 mimics
543m	miR-543 mimics
mNC	mimics negative control
758I	miR-758 inhibitor
543I	miR-543 inhibitor
INC	inhibitor negative control
3′UTR	3′ untranslated region
WT	wild type
MUT	mutant
RT-PCR	real time-PCR
ANOVA	one-way analysis of variance
MOI	multiplicity of infection
MADS	MCM1, Agamous, Deficiens, Serum Response factor
*LPGAT1*	iysophosphatidylglycerol acyltransferase 1
*DCP1A*	decapping mRNA 1A
*RUNX1T1*	RUNX1 translocation partner 1
*EIF4B*	eukaryotic translation initiation factor 4B
*KLF12*	kruppel like factor 12
*PURB*	purine rich element binding protein B
SDS-PAGE	sodium dodecyl sulfate polyacrylamide gel electrophoresis
PBS	phosphate buffer solution
DAPI	4′,6-diamidino-2-phenylindole
PVDF	polyvinylidene fluoride
*PPP2R1B*	protein phosphatase 2 scaffold subunit A, beta
*PPP2R2A*	protein phosphatase 2 regulatory subunit B, alpha
*PPP2R2B*	protein phosphatase 2 regulatory subunit B, beta
*PPP2R5A*	protein phosphatase 2 regulatory subunit B′ alpha
*PPP2R5E*	protein phosphatase 2 regulatory subunit B′ epsilon
*PPP2R3A*	protein phosphatase 2 regulatory subunit B″ alpha
MRFs	myogenic regulatory factors
*MYOD1*	myogenic differentiation 1
*MYOG*	myogenin
*MYF5*	myogenic factor 5
*MYF6*	myogenic regulatory factor 4
*GAPDH*	glyceraldehyde-3-phosphate dehydrogenase
