# Supplementary material for: MEF2A Regulates the MEG3-DIO3 miRNA Mega Cluster-Targeted PP2A Signaling in Bovine Skeletal Myoblast Differentiation
Source: Int J Mol Sci. 2019 Jun 4;20(11):2748. doi: 10.3390/ijms20112748 (PMC6600538; doi:10.3390/ijms20112748)
Supplement: Supplementary file 1 [file ijms-20-02748-s001.zip › ijms-510290-SI/Table S1.pdf]

Table S2 Information of the MEG3-DIO3 locus on chromosome 21 in the bovine genome.

Gene	Location	Gene types	Product
MEG3	67377727..67395997	lncRNA	lncRNA
bta-miR-493	67416605..67416693	miRNA	bta-miR-493
bta-miR-665	67423449..67423520	miRNA	bta-miR-665
RTL1	67427514..67431509	protein-coding gene	RTL
Peg11AS	67427670..67428587	lncRNA	PEG11 antisense RNA/Anti-Rtl1
bta-miR-431	67427770..67427883	miRNA	miR431
bta-miR-433	67428646..67428769	miRNA	miR433
bta-miR-127	67429744..67429838	miRNA	miR127
bta-miR-432	67431234..67431323	miRNA	miR432
bta-miR-136	67431418..67431509	miRNA	miR136
LOC100847413	67440654..67556867	lncRNA	uncharacterized LOC100847413
bta-miR-370	67457237..67457311	miRNA	bta-miR-370
bta-miR-379	67561869..67561954	miRNA	bta-miR-379
bta-miR-411a/miR-411	67563098..67563179	miRNA	bta-miR-411a
bta-miR-299	67563570..67563632	miRNA	bta-miR-299
bta-miR-380	67564803..67564863	miRNA	bta-miR-380
bta-miR-411b	67564957..67565031	miRNA	bta-miR-411b
bta-miR-1197	67565299..67565395	miRNA	bta-miR-1197
bta-miR-323	67565468..67565553	miRNA	bta-miR-323
bta-miR-758	67565766..67565853	miRNA	bta-miR-758
bta-miR-329b	67566507..67566589	miRNA	bta-miR-329b
bta-miR-329a	67566817..67566899	miRNA	bta-miR-329a
bta-miR-494	67569676..67569760	miRNA	bta-miR-494
bta-miR-1193	67570008..67570112	miRNA	bta-miR-1193
bta-miR-543	67571825..67571904	miRNA	bta-miR-543
bta-miR-495	67573235..67573315	miRNA	bta-miR-495
bta-miR-154c	67575252..67575326	miRNA	bta-miR-154c
bta-miR-376e	67577941..67578022	miRNA	bta-miR-376e
bta-miR-376c	67578312..67578397	miRNA	bta-miR-376c
bta-miR-376d	67578686..67578767	miRNA	bta-miR-376d
bta-miR-654	67578839..67578919	miRNA	bta-miR-654
bta-miR-376b	67579062..67579154	miRNA	bta-miR-376b
bta-miR-376a	67579421..67579519	miRNA	bta-miR-376a
bta-miR-1185	67581370..67581455	miRNA	bta-miR-1185

Gene name	Location/ mature miRNA	Gene types	Product
bta-miR-3956	67582109..67582206	miRNA	bta-miR-3956
bta-miR-381	67583073..67583147	miRNA	bta-miR-381
bta-miR-487b	67583558..67583641	miRNA	bta-miR-487b
bta-miR-539	67584372..67584449	miRNA	bta-miR-539
bta-miR-544a	67586358..67586448	miRNA	bta-miR-544a
bta-miR-655	67587315..67587411	miRNA	bta-miR-655
bta-miR-411c	67589478..67589551	miRNA	bta-miR-411c
bta-miR-487a	67589864..67589943	miRNA	bta-miR-487a
bta-miR-3578	67592873..67592958	miRNA	bta-miR-3578
bta-miR-382	67592878..67592953	miRNA	bta-miR-382
bta-miR-134	67593257..67593329	miRNA	bta-miR-134
bta-miR-485	67593955..67594027	miRNA	bta-miR-485
bta-miR-453	67594686..67594765	miRNA	bta-miR-453
bta-miR-154a	67598039..67598122	miRNA	bta-miR-154a
bta-miR-154b	67598357..67598437	miRNA	bta-miR-154b
bta-miR-496	67598869..67598969	miRNA	bta-miR-496
bta-miR-377	67600490..67600558	miRNA	bta-miR-377
bta-miR-541	67602344..67602427	miRNA	bta-miR-541
bta-miR-3957	67602730..67602806	miRNA	bta-miR-3957
bta-miR-409a	67603246..67603324	miRNA	bta-miR-409a
bta-miR-409b	67603246..67603324	miRNA	bta-miR-409b
bta-miR-412	67603388..67603477	miRNA	bta-miR-412
bta-miR-369	67603541..67603609	miRNA	bta-miR-369
bta-miR-410	67603865..67603945	miRNA	bta-miR-410
bta-miR-656	67604689..67604767	miRNA	bta-miR-656
LOC104975448	67771226..67774818	lncRNA	uncharacterized LOC104975448
LOC100296203	67948388..68038550	lncRNA	uncharacterized LOC100296203
LOC101907771	68047232..68086647	lncRNA	uncharacterized LOC101907771
LOC104975449	68072260..68110907	lncRNA	uncharacterized LOC104975449
bta-miR-1247	68149435..68149512	miRNA	bta-miR-1247
DIO3	68150464..68152565	protein-coding gene	DIO3
