# Supplementary material for: MEF2A Regulates the MEG3-DIO3 miRNA Mega Cluster-Targeted PP2A Signaling in Bovine Skeletal Myoblast Differentiation
Source: Int J Mol Sci. 2019 Jun 4;20(11):2748. doi: 10.3390/ijms20112748 (PMC6600538; doi:10.3390/ijms20112748)
Supplement: Supplementary file 1 [file ijms-20-02748-s001.zip › ijms-510290-SI/Table S3.pdf]

Table S1. Summary information of the genes used for RT-PCR in this study

Gene	GenBank ID	Forward primer sequence (5′-3′)	Reverse primer sequence (5′-3′)	Product
GAPDH	NM_001034034	AGTTCAACGGCACAGTCAAGG	ACCACATACTCAGCACCAGCA	124bp
MEG3	NR_037684	GCATAGAAGAGGCGGTCAGTAA	AAACGAGAAGCGTGAGACAGG	92bp
PPP2R2A	NM_001191533	ATAACCCTGGAAGCATCACGA	CATCTTTCTTTCGCTTGCCACT	91bp
PPP2R2C	XM_024993627	AGGGAGCAGTTTCATCCACCT	GTGTGGTTGAACTCGACGGTA	103bp
PPP2R3A	XM_024996309	TACAGAAAGAGGGACGAATGAGC	CATCCATACAGCGGAACCAG	108bp
PPP2R5A	NM_001081728	GCTTCACCCGGAAATCGGTG	TTGAGTTGGGGCAAGGGGTG	112bp
MYOD1	NM_001040478	AACCCCAACCCGATTTACC	CACAACAGTTCCTTCGCCTCT	196bp
MYOG	NM_001111325	GGCGTGTAAGGTGTGTAAG	CTTCTTGAGTCTGCGCTTCT	85bp
MYF5	NM_174116	CCAGCACCGATTCTCAACCT	CAGGTTGTCTTGCTTTGGGG	151bp
MYF6	NM_181811	GTGATAACTGCCAAGGAAGGAG	CGAGGAAATGCTGTCCACGA	93bp
